# Improving the Response of Accelerometers for Automotive Applications by Using LMS Adaptive Filters: Part II

**DOI:** 10.3390/s100100952

**Published:** 2010-01-26

**Authors:** Wilmar Hernandez, Jesús de Vicente, Oleg Y. Sergiyenko, Eduardo Fernández

**Affiliations:** 1 Department of Circuits and Systems, EUIT de Telecomunicación, Universidad Politécnica de Madrid (UPM), Campus Sur UPM, Ctra. Valencia km 7, Madrid 28031, Spain; 2 Department of Applied Physics, ETSI Industriales, Universidad Politécnica de Madrid, Calle José Gutierrez Abascal 2, Madrid 28006, Spain; E-Mail: jvo@etsii.upm.es; Tel.: +34-91-336-3125; Fax: +34-91-336-3000; 3 Engineering Institute of Autonomous, University of Baja California, Mexicali, Baja California, México; E-Mail: srgnk@iing.mxl.uabc.mx; 4 EUIT de Telecomunicación, Universidad Politécnica de Madrid (UPM), Campus Sur UPM, Ctra. Valencia km 7, Madrid 28031, Spain; E-Mail: edfernan@alumnos.euitt.upm.es

**Keywords:** piezoresistive accelerometer, conventional LMS adaptive filter, fast LMS adaptive filter

## Abstract

In this paper, the fast least-mean-squares (LMS) algorithm was used to both eliminate noise corrupting the important information coming from a piezoresisitive accelerometer for automotive applications, and improve the convergence rate of the filtering process based on the conventional LMS algorithm. The response of the accelerometer under test was corrupted by process and measurement noise, and the signal processing stage was carried out by using both conventional filtering, which was already shown in a previous paper, and optimal adaptive filtering. The adaptive filtering process relied on the LMS adaptive filtering family, which has shown to have very good convergence and robustness properties, and here a comparative analysis between the results of the application of the conventional LMS algorithm and the fast LMS algorithm to solve a real-life filtering problem was carried out. In short, in this paper the piezoresistive accelerometer was tested for a multi-frequency acceleration excitation. Due to the kind of test conducted in this paper, the use of conventional filtering was discarded and the choice of one adaptive filter over the other was based on the signal-to-noise ratio improvement and the convergence rate.

## 1. Introduction

This paper was written as a continuation of [1]. In [1], there were two things left for further analysis. The first one was to test the piezoresistive accelerometer under test for the case in which the excitation acceleration had multiple frequency components; and the second one was to try to improve the convergence rate of the optimal adaptive filter by using another filter of the same family of LMS filters.

Testing the accelerometer for a multi-frequency acceleration excitation is very important because car manufacturers should know the dynamic response of the sensor systems they have embedded in the cars. Each specific application requires its specific kind of accelerometer. Some examples of applications are low frequency monitoring, vibration sensing, motion analysis, tilt, safety crash testing, shock testing, off-road testing, road testing, and so on.

Car manufactures should know the disturbance rejection of the sensor systems embedded in cars and how they respond to multi-frequency excitations, distinguishing two or more very close relevant signals from noise and/or interferences, because in real-life driving conditions we do not know the exact value of the frequency of interest, and the characteristics of disturbances and noise signals either.

So, if designers use fixed filters (*i.e.*, Butterworth, Chebyshev, Bessel, *etc.*), which is what currently happens, they are bound to let noise pass through the sensor systems. Therefore, in today’s cars, in order to diminish this problem, designers use several parallel systems and auxiliary electronic circuits, which makes the system expensive, so that the filtering problem does not rely completely on only one filter.

Solving the above mentioned filtering problem can save lives in car accidents, because accelerometer are widely used in the airbag deployment system, in the anti-lock breaking system and in the active suspension system, among others.

Also, improving the convergence rate of the sensor systems embedded in cars, when filtering noise and interferences, is of paramount importance for drivers and passengers, because it means that such systems can respond very fast to unpredictable situations when driving under very difficult conditions that can cost human lives.

In the scientific literature on instrumentation and signal treatment for sensors, several research works on the application of classical and advanced filtering techniques aimed at improving the performance of sensor systems have been reported [1,2]. Authors have used robust algorithms [3,4], classical filters [5,6] and classical signal conditioning techniques [7,8].

Here, as in the first part of this research work [1], easy and inexpensive adaptive filtering techniques [9,10] have been used to improve the performance of the piezoresistive accelerometer 1201F of the manufacturer Measurement Specialties. The characteristics of the accelerometer were already mentioned in [1] and more general information about accelerometers can be found in [7,8,11].

This part of the research work was aimed at testing the above accelerometer for a multi-frequency acceleration excitation by using both the same LMS algorithm as in [1] and a fast LMS algorithm. Finally, based on the experimental results, a decision was made about what algorithm was best for solving the problem at hand.

## 2. Adaptive Filtering

The problem was to estimate a signal buried in a broad-band noise background, where we had little information of the signal and noise characteristics. Also, the relevant signal was a multi-frequency acceleration excitation and the noise reduction was treated as an unknown signal estimation problem, a condition that justified the fact that the use of fixed filters was discarded.

Therefore, in order to satisfactorily solve the previously outlined problem, it should be pointed out that the chosen adaptive filter should fulfil the following design requirements [10]:

1. It should not have a high computational burden.
2. It should have good numerical properties, rate of convergence and round-off error rejection.
3. It is required to yield good transient and tracking performance, disturbance rejection and robustness.

However, as we demand more requirements, the designed filter has to be more complex. This fact led us to make a trade-off between the attributes that the filter should have and the final performance requirements.

According to the above statements, both the conventional and the fast LMS adaptive filters were chosen to carry out the filtering process in this research. The use of the LMS adaptive filter seemed to be one of the best solutions because of its robustness (its model-independent property) [9,10].

Also, due to its low round-off errors, stability characteristics and easy implementation, the LMS adaptive filter is well suited in applications where we have to design systems for continuous operation without any human intervention.

This filter was satisfactorily used in the first part of this research [1]. However, in order to improve the convergence rate and the signal-to-noise ratio (SNR) for a better performance of the accelerometer when placed in cars, it was necessary to test a fast LMS adaptive filter, which performs the adaptation of the filter parameters in the frequency domain.

In accordance with [10], there are two main reasons for seeking adaptation in the frequency domain:

1. Frequency-domain adaptive filters can deal with the requirement of long memory satisfactorily providing good solutions to the computational complexity problem.
2. A more uniform convergence rate is achieved by taking advantage of the orthogonality properties of the discrete Fourier transform (DFT) and related discrete transforms.

Basically, the structure of the fast LMS adaptive filter is the one of a block-adaptive filter. The input signal is divided into several blocks of the same length by using a serial-to-parallel converter, and the resulting blocks of this conversion are filtered by a finite impulse response (FIR) filter, one block of data samples at a time. The adaptive process begins and continue on a block-by-block basis. In fact, the filter parameters are adapted in the frequency domain by using the fast Fourier transform (FFT) algorithm [12–15].

**Figure 1.**
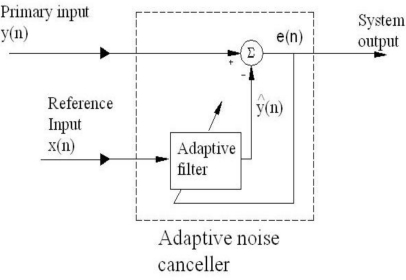
Schematic diagram of the ANC.

According to Haykin [10], it is known that the overlap-save method and the overlap-add method provide two efficient procedures for fast convolution – that is, the computation of linear convolution using the DFT. In this paper the fast LMS algorithm based on overlap-save sectioning (assuming real-valued data) [16] was used in an adaptive noise canceller (ANC) device [9,10]. Figure 1 shows the schematic diagram of such a device and a summary of this algorithm is given next.

*A summary of the fast LMS adaptive filter. From Shynk* [16] *and Haykin* [10]

*Initialisation*:

**Ŵ**(0) = 2*M*-by-1 null vector, where Ŵ is the frequency-domain tap-weight vector of the FIR filter for the *k*th block of input data and *M* is the length of the FIR filter.
*P_i_*(0) = *δ_i_*, *i* = 0, …, 2*M* – 1, where *P_i_* is an estimate of the average power in the *i*th bin

*Notations*:

**0** = *M*-by-1 null vector
FFT = fast Fourier transformation
IFFT = inverse fast Fourier transformation
α = adaptation constant
γ is a *forgetting factor* that controls the effective “memory” of the iterative process, this is a constant chosen in the range 0 < *γ* < 1

*Computation*: For each new block of *M* input samples, compute

**U**(*k*) = diag{FFT[*u*(*kM* − *M*),..., *u*(*kM* − 1), *u*(*kM*),...,*u*(*kM + M* − 1)]*^T^*}
**y**(*k*) = last *M* elements of IFFT[**U**(*k*)**Ŵ**(*k*)]
**e**(*k*) = **d**(*k*) – **y**(*k*)
  $$E\left(k\right)=\mathrm{FFT}\left[\begin{array}{c} 0\\ e\left(k\right) \end{array}\right]$$
*P_i_*(*k*) = γ*P_i_*(*k*−1) + (1 − γ)| *U_i_*(*k*)|^2^, *i* = 0, 1, …, 2*M* − 1
  $$D(k)=\text{diag}[P_{0}^{-1}(k),\;P_{1}^{-1}(k),\;\ldots ,\;P_{2M-1}^{-1}(k)]$$
**φ** (*k*) = first *M* elements of IFFT[**D**(*k*)**U***^H^*(*k*)**E**(*k*)]
**Ŵ**(*k* + 1) = **Ŵ**(*k*) + αFFT $\left[\begin{array}{c} \varphi (k)\\ 0 \end{array}\right]$

## 3. Results of the Experiment

**Figure 2.**
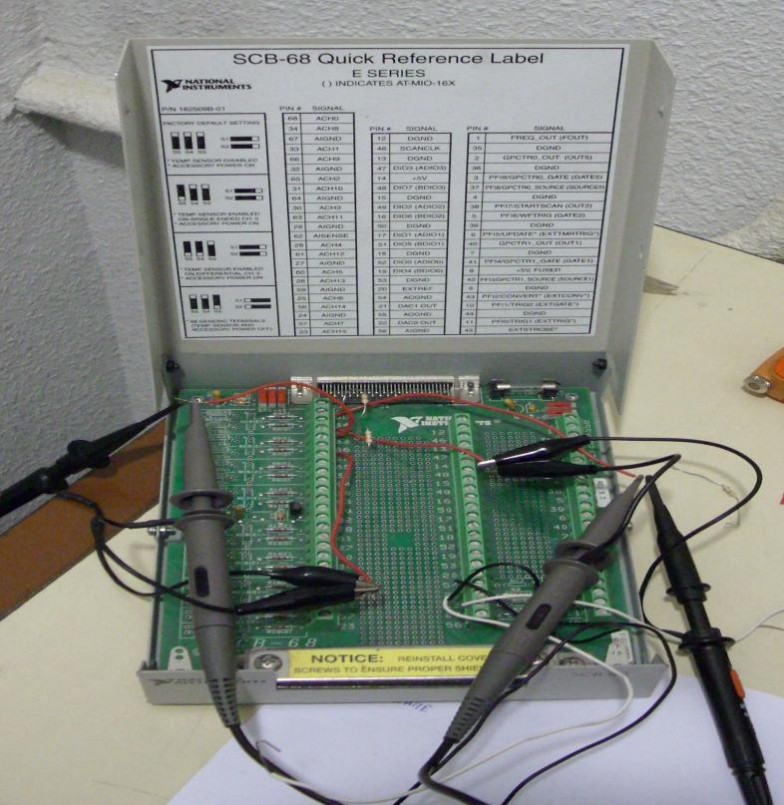
NI SCB-68 connector block.

**Figure 3.**
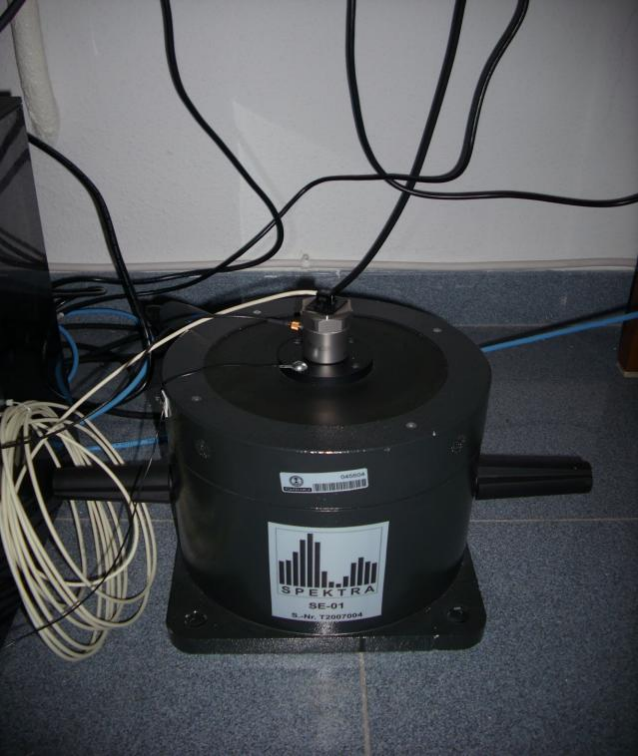
Vibration exciter SE-1.

**Figure 4.**
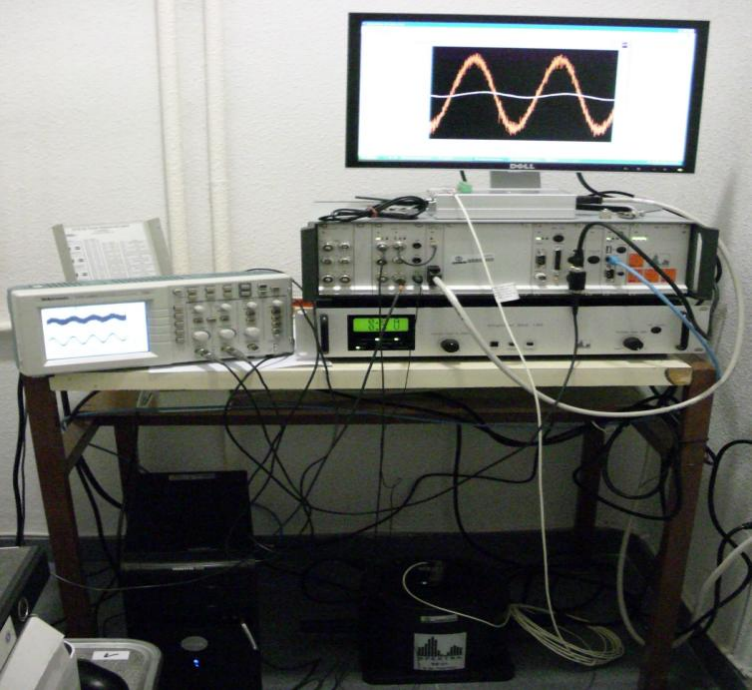
Experimental setup: vibration control system SRS-35, power amplifier PA-14-180, vibration exciter SE-1, and Standard-PC.

As in [1], in the experiment, the accelerometer 1201F-1000-10-240X (Model 1201F, 1,000 g Full Scale Range, 10 VDC excitation, 240 inches cable, and no options), was tested under laboratory conditions by using the CS18 TF calibration system (SPEKTRA). This system can carry out calibrations of sensors with/without amplifiers in the frequency range 3 Hz to 5 kHz, with a repeatability of the calibration under identical conditions up to 5 kHz better than 0.5%. Here, the accelerometer 1201F-1000-10-240X was tested with a multi-frequency acceleration excitation of maximum amplitude 2 g and frequency components 200 Hz, 500 Hz and 1 kHz. The National Instruments Data Acquisition Card NI DAQCard-6062E was used for the laboratory experiments. In addition, for the multi-frequency acceleration excitation experiment the sampling frequency was 100 kHz. Figure 2 shows the National Instruments 68-pin shielded desktop connector block (NI SCB-68) DAQ device used in the laboratory experiment, Figure 3 shows the vibration exciter and Figure 4 shows the overall experimental setup.

**Figure 5.**
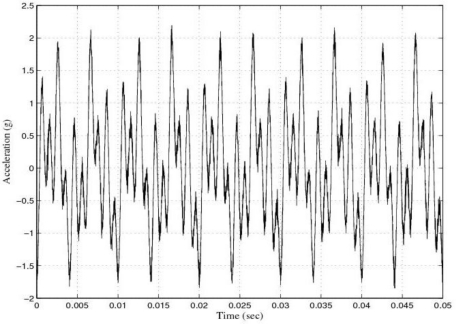
Response of the sensor system before filtering for a multi-frequency acceleration of maximum amplitude 2 g and frequencies 200 Hz, 500 Hz and 1 kHz: Time waveform.

**Figure 6.**
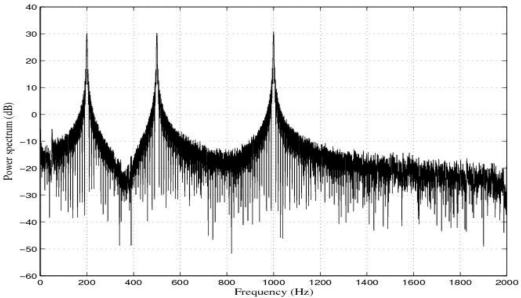
Response of the sensor system before filtering for a multi-frequency acceleration of maximum amplitude 2 g and frequencies 200 Hz, 500 Hz and 1 kHz: Power spectrum (dB).

The response of the sensor before filtering for the experimental tests at 50 Hz, 100 Hz, 200 Hz, 500 Hz and 1 kHz was shown in [1]. Also in [1] the filtering process for the above tests was carried out by using 4-order band-pass digital Butterworth filters and a LMS adaptive filter. Figure 5 shows the response of the sensor for the multi-frequency acceleration excitation and Figure 6 shows the power spectrum of such a signal.

As in [1], the parameters of the LMS adaptive filter were the following: a tap-weight vector of length *M* equal to 100, and a step-size parameter *μ* equal to 1 over the maximum value of the power of the tap-input vector **x**(*n*) [10].

**Figure 7.**
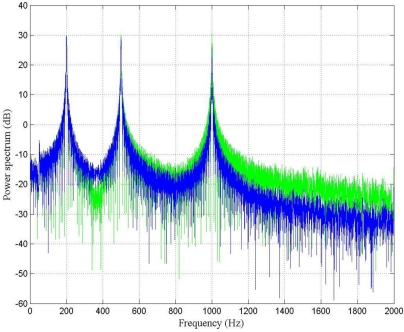
Power spectrum (dB) of the output signal before (green) and after (blue) filtering by using the LMS adaptive filter.

Figure 7 shows the power spectrum of the output signal before and after filtering by using the LMS adaptive filter. It is important to point out that at 200 Hz the LMS adaptive filter does not perform very well; however, the higher the frequency, the better the SNR.

**Figure 8.**
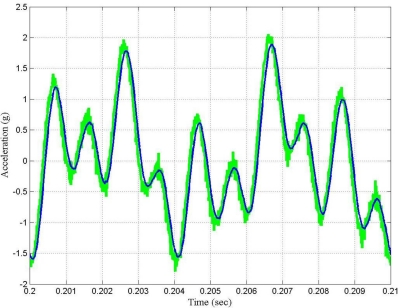
Time waveforms of the output signal for the case under test: Green—output signal before filtering; Blue—output signal after filtering by using the LMS adaptive filter.

Figure 8 shows the time waveform of the output signal before filtering and after filtering by using the LMS adaptive filter. From Figures 7 and 8, it can be said that in general the performance of the LMS adaptive filter was satisfactory.

**Figure 9.**
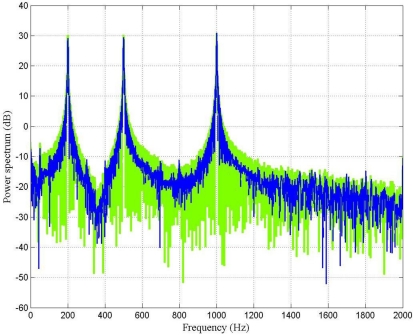
Power spectrum (dB) of the output signal before (green) and after (blue) filtering by using the fast LMS adaptive filter.

Figure 9 shows the power spectrum of the output signal before and after filtering by using the fast LMS adaptive filter with the following parameters: *M* = 100, α = 0.1, *δ_i_* = 0.1, and γ = 0.999. From this figure it can be seen that the performance of the filter was satisfactory at every frequency of analysis.

**Figure 10.**
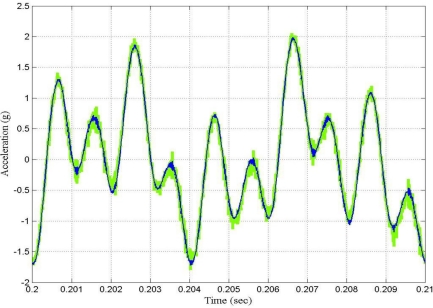
Time waveforms of the output signal for the case under test: Green—output signal before filtering; Blue—output signal after filtering by using the fast LMS adaptive filter.

**Figure 11.**
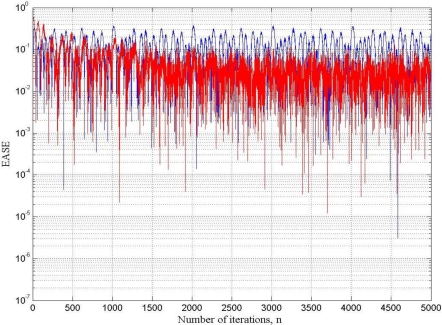
Learning curve of the conventional (blue) and the fast (red) LMS adaptive filters for the case under test: EASE is the ensemble-average squared error (logarithmic scale).

Figure 10 shows the time waveform of the output signal before filtering and after filtering by using the fast LMS adaptive filter, and Figure 11 shows the learning curves of both the conventional and the fast LMS adaptive filter.

From Figures 7, 9 and 11, it can be seen that the performance of the fast LMS adaptive filter was better than one of the conventional LMS adaptive filter. For the case under test, the conventional LMS adaptive filter behaved worst, it exhibited the worst SNR and the slowest rate of convergence.

## 4. Conclusions

In this paper, a real-life filtering problem of multi-frequency acceleration excitation to test an accelerometer for automotive applications has been solved by using both a conventional and a fast LMS adaptive filter. The results of the experiment were satisfactory for both filters and it has been shown that the best option to carry out the filtering problem discussed in this paper was to use the fast LMS adaptive filter.
